# Uganda’s evolving national biosafety system: lessons from the past 30 years

**DOI:** 10.3389/fbioe.2025.1654335

**Published:** 2025-10-20

**Authors:** B. M. Zawedde, M. Kwehangana, I. Ongu, A. W. Ibanda, P. Wasswa, A. Kiggundu, C. Mugoya, D. Kasule, A. M. Makara, D. Hafashimana, T. Ssengooba, H. K. Oloka

**Affiliations:** ^1^ Secretariat, National Agricultural Research Organization (NARO), Entebbe, Uganda; ^2^ Research Management and Quality Assurance, Uganda National Council for Science and Technology, Kampala, Uganda; ^3^ Science Foundation for Livelihoods and Development, Kampala, Uganda; ^4^ Uganda Biotechnology and Biosafety Consortium, Kampala, Uganda; ^5^ College of Agriculture and Environmental Sciences, Makerere University, Kampala, Uganda; ^6^ National Livestock Resources Research Institute, NARO, Wakiso, Uganda; ^7^ Target Malaria Project, Uganda Virus Research Institute, Entebbe, Uganda; ^8^ Corporate Affairs and International Collaborations, Uganda National Council for Science and Technology, Kampala, Uganda; ^9^ Enviro-Impact and Management Consults, Kampala, Uganda; ^10^ Atiri Agri-Bio Solutions, Kampala, Uganda

**Keywords:** biotechnology regulation, GMOs, competent authority, africa, science outreach

## Abstract

Uganda has made progress towards developing a functional biosafety system. The system has evolved in the past three decades to enable substantial application of modern biotechnology in different sectors. Key informant interviews were used to capture tacit knowledge from respondents who were identified to have vast knowledge and experience of the biosafety system of Uganda in the past 30 years. Secondary data was then used to fill the gaps in the knowledge map. From the findings we were able to identify the key drivers of policy reforms that shaped the evolution of the biosafety regulatory system; policy, institutional developments, partnerships, public participation and engagements milestones that contributed to developing the biosafety system in Uganda. We discuss the lessons learnt and their implications for on-going and future biosafety policy and legal discourse. We share some strategic recommendations that we believe if implemented will enable Uganda, and other developing countries, to put in place a coordinated and evidence-based regulatory system, which is required for effective application and adoption of the current and emerging biotechnologies. Uganda’s case study is also a learning experience for countries that are in the process of establishing biosafety frameworks.

## 1 Introduction

Modern biotechnology has been applied in different aspects of our lives, including agriculture, healthcare, environmental management, and industrial development (Gupta et al., 2017; Lokko et al., 2017). The first application of the genetic engineering (GE) tools was developed in 1973 by Herbert Boyer and Stanley Cohen, who created a bacterium resistant to the antibiotic Kanamycin (Wolff and Lederberg, 1994). Since then, the world has benefited from new or improved medical innovations such as vaccines, hormones and proteins; improved livestock breeds and crop varieties; environmental remediation products, biofuels; and industrial home care products, such as detergents (Ma, 2021; Ghosh, 2024). The advancements and use of GE tools and products have occurred in tandem with the development of enabling biosafety regulatory frameworks to address potential risks associated with the technologies.

Biosafety regulatory frameworks encompassing policies, institutional capacities and public participation to ensure the safe development and use of GE tools continue to evolve in the world. As new scientific techniques are developed, countries advance their regulatory capacities to keep up with these developments. While GE applications in healthcare have been largely regulated under existing pharmaceutical rules, many countries established a separate legal and regulatory system to guide research, development and use of the technology in agriculture. This partially stems from decisions adopted under the Convention on Biological Diversity-CBD (1992), the Cartagena Protocol on Biosafety-CPB, (2001) and the Nagoya-Kuala Lumpur Supplementary Protocol on Liability and Redress (2010). Under the purview of decisions of these global instruments, African countries were encouraged to develop their respective biosafety systems specific to their country needs and priorities. A lot of progress has been made in this regard, but countries are at different stages of developing regulatory systems for GE.

The progress with developing a policy and regulatory system for GE technology depended on different factors. As technology advances further with newer tools such as genome editing, regulators also have had to rethink and review their existing biosafety systems to conform with the advances. Within the East African Community (EAC) region, countries have adopted different approaches in developing their regulatory frameworks. Tanzania, for example, established a regulatory framework based on its environmental law while Kenya and Rwanda on the other hand approved standalone legislation on biosafety, done albeit more than a decade apart (Government of Kenya, 2009; Government of Rwanda, 2024). Kenyan regulators further established guidelines that address emerging gene technologies such as genome editing. Uganda, Kenya Rwanda, and Tanzania have all tested GE crops under field conditions, with Kenya further approving the cultivation of GE cotton, cassava, and maize (Ongu et al., 2023). Burundi, the Democratic Republic of Congo and South Sudan are yet to develop specific laws on biosafety and there are no reported field experiments with GE crops.

Uganda has made progress towards developing a functional biosafety system. The system has evolved in the past three decades to enable field experiments and the importation of processed GE products in the country. As opposed to other African countries that readily approved biosafety laws, Uganda is yet to enact a standalone biosafety legislation, despite decades of efforts. Several factors may have impacted this apparent delay in enacting a standalone biosafety law. It is therefore important to unpack these factors and understand the evolution of Uganda’s biosafety system and provide context to policymakers, researchers, product developers, regulators and consumers. Uganda’s case study may serve as a learning experience for countries that are in the process of establishing biosafety frameworks from the application, development and possible uptake of GE products. This paper analyses the journey to development of a biosafety system in Uganda that has enabled substantial application of modern biotechnology in different sectors.

## 2 Objectives

This study examines the factors that have shaped the evolution of the biosafety regulatory framework in Uganda. The objectives of the study were to:

a. Understand the key drivers of policy reforms for regulation for modern biotechnologies and their products.
b. Analyse the key milestones in developing the biosafety system in Uganda
c. Explore lessons learnt and their implications for national biosafety systems discourses.

## 3 Conceptual framework

**FIGURE 1:**
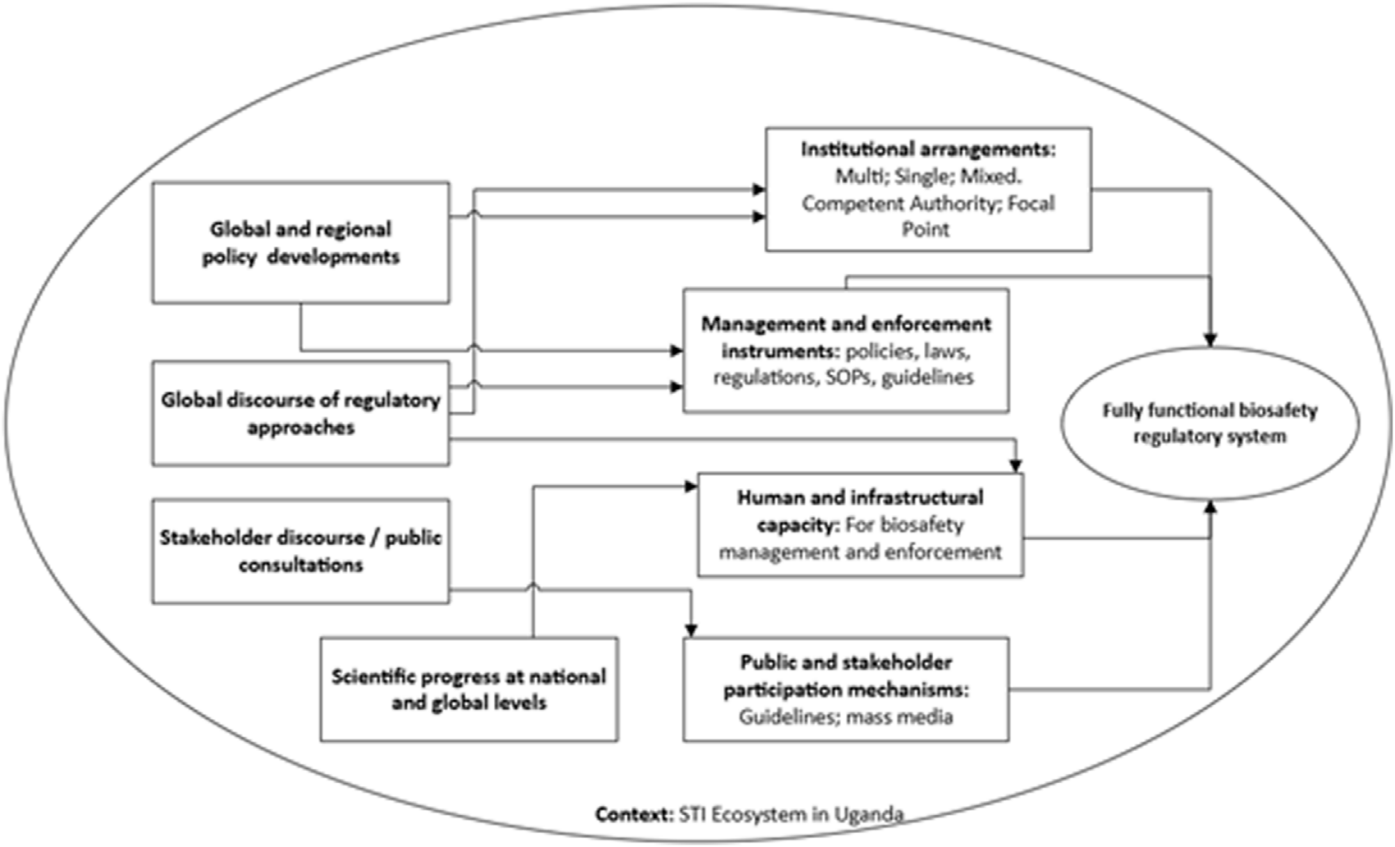
Conceptual framework for the evolution of biosafety regulation in Uganda (developed by the authors).

Biosafety in Uganda is largely interpreted as regulation of modern biotechnology for its safe development, transfer application and use (Government of Uganda, 2008). Policy and regulatory discourse in the past 30 years have nonetheless focused on safety of genetic engineering (GE) technologies in crop and livestock agriculture as well as applications for human health. Establishment of the National Biosafety Framework entailed putting in place key requirements to have a fully functional biosafety system (Figure 1). These included: a national biosafety policy; a law and attendant regulations; guidelines and standard operating procedures; enforcement procedures; public participation mechanisms; and regulatory institutions (Nyiira et al., 2000). This paper reviews the progress towards attaining the ‘ideal’ set of requirements that would enable a functional system that supports biosafety management during research, development, and environmental/commercial release of GE technologies. The paper further examines the factors that influenced the country in taking specific policy decisions and provides some insights into future efforts.

Uganda’s biosafety framework is an evolving system designed to protect human health, the environment, and biodiversity while fostering the responsible use of modern biotechnology techniques. Through policies and legislations, sector-specific regulations, institutional coordination, capacity building and public participation, the country is working to create a model or ‘perfect’ biosafety environment. We explore the extent of progress made towards addressing each of these pillars in the biosafety system and which specific factors have contributed to the various policy and institutional milestones over the past three decades.

## 4 Methodology

Three approaches were used in this paper: a qualitative study, literature review and co-authorship selection. Authorship identification was strategic to bring on board a consortium of experts from different institutions who have varied but in-depth experience about the development of the biosafety system in Uganda. The qualitative study used key informant interviews (KIIs) with respondents who were identified to have vast knowledge and experience (Donnelly et al., 2023) of the biosafety system of Uganda in the past 30 years. This methodology was selected as it focuses on the knowledge of the expert and allows an in-depth description of context and practices, sharing experiences and obtaining perceptions (Gabor, 2017) of the predicted or desirable future biosafety system in the country. Purposive sampling technique was used to select twenty (20) key informants from Government research and regulatory agencies, academia, private sector and civil society who participated in Uganda’s biosafety system at different stages, including the formulation (technical leaders), operationalization (regulators) or compliance (scientists and researchers). Tacit knowledge was captured from the key informants using open-ended questions to guide storytelling in face-to-face interviews. Audio-visual tools were used and informed consent was obtained before the interview. The captured stories were compiled into explicit knowledge. The explicit knowledge was arranged into recurring themes and insights that were used to contextualize and map the presentation of the findings.

In addition to the interviews, secondary data was collected using literature review, synthesized, and corroborated with the KIIs’ data to fill the gaps in the knowledge map. This included a review of Uganda National Council for Science and Technology (UNCST) decision documents for biosafety applications received since the formation of the National Biosafety Committee in 1996. Co-authorship of this paper was based on selection of experienced experts from government regulatory and research agencies, as well as civil society who have been involved in Uganda’s biosafety system at different stages of development and evolution.

## 5 Findings

The study findings are represented under three (3) thematic areas: key drivers of policy reforms that shaped the evolution of the biosafety regulatory system; key milestones in developing the biosafety system in Uganda; and lessons learnt and their implications for national biosafety systems discourses. Drawing from these findings, some strategic recommendations are presented for consideration by those involved in developing and implementing national biosafety system.

### 5.1 Key drivers of policy reforms that shaped the evolution of the biosafety regulatory system

The biosafety system in Uganda has evolved within a broader context of science, technology and innovations regulatory oversight in and outside the country. Policy and regulatory reforms evolved alongside technology advancements, environmental laws, and emerging societal challenges such as human and agricultural pests and diseases, climate change impacts, famine, and other emergencies. Policy and regulatory advancements in the country largely started in the early 1990s in response to local and international needs. The main drivers of these policy changes are highlighted below.

#### 5.1.1 Early developments in science regulation, and oversight in Uganda

As far back as 1965, the government of Uganda materialized the support for science, technology and innovation (STI) having predicted the role it would play in the growth of the economy of Uganda. The National Research Council (NRC), established in 1970, was mandated to coordinate research in key sectors of the economy (Jjagwe et al., 2024). The NRC was initially housed within the Ministry of Planning but was later placed as a department within the Office of the President. Research coordination under the NRC was conducted through different research committees for industrial research, social and natural sciences. This approach was not directly linked to the various sectors and ministries that directly conducted research or needed products from research, and as such, the government, through a Statute (Chapter 211 of the Laws of the Republic of Uganda), established the Uganda National Council for Science and Technology (UNCST) in 1990. The mandate of UNCST is to develop and implement strategies for integrating science and technology (S&T) into Uganda’s national development process. The UNCST retained some functions of the NRC but integrated new roles in supporting all sectors in formulating and supporting the implementation of sector-specific STI policies. A key function of UNCST under the UNCST Act (1990) was to act as a clearing house for all scientific research and development undertakings in Uganda (Government of Uganda, 1990). This function directly placed UNCST at the forefront of regulating key scientific advances, including modern biotechnology as each research activity required prior approval by the Council.

#### 5.1.2 Global and regional policy influence on Uganda’s biosafety policy developments

The increased interest and emergence of global environmental and biodiversity regulatory systems are considered key drivers to Uganda’s biosafety policy reforms. Uganda sent delegates to the Earth Summit in Rio in 1992 and ratified the Convention on Biological Diversity (CBD) in 1993. Article 8 (g) of the Convention requires parties to “establish or maintain means to regulate, manage or control the risks associated with the use and release of living modified organisms resulting from biotechnology, which are likely to have adverse environmental impacts, that could affect the conservation and sustainable use of biological diversity, taking also into act the risks to human health”. In addition, Article 19 Section 3 of the Convention, required contracting parties to “consider the need for and modalities of a Protocol setting out appropriate procedures, including, particular, advance informed agreement, in the field of the safety transfer, handling and use of any living modified organisms resulting from biotechnology that may have adverse effects on the conservation and sustainable use of biological diversity”. This process culminated into the Cartagena Protocol, adopted in 2000. Uganda ratified the Cartagena Protocol on Biosafety in November 2001, indicating commitment to developing a clear system for addressing biosafety issues. The Nagoya-Kuala Lumpur Supplementary Protocol on Liability and Redress to the Cartagena Protocol on Biosafety was later adopted in 2010, with Uganda acceding to it in June 2014.

Parties to the CPB were obliged to establish key legal, administrative (institutional) and other mechanisms to receive applications, conduct risks assessments, and share information through the clearing house mechanism for information exchange on GE developments in the country. Uganda therefore designated a National Focal Point (both for general Biosafety and for the Clearing House Mechanism), within the Ministry responsible for environment and UNCST as the Competent National Authority for purposes of domesticating and implementation of the CPB. Formal appointment of the two administrative and regulatory structures was a major policy decision, directly driven by the global adoption and the country’s subsequent ratification of the CBD and CPB. In addition to environmental treaties, Uganda has also been a member of Codex Alimentarius Commission since 1964. The Commission, a joint effort between the Food and Agriculture Organization and the World Health Organization of the United Nations (UN), publishes food safety standards that have been largely adopted by member countries. Standards for food safety assessment for GMOs are largely guided by Codex standards. In 2011, Uganda published the *Guidelines for the interpretation of data for food safety of foods derived from recombinant-DNA plants*. These guidelines build on guidance under the Codex Alimentarius.

Regional harmonisation efforts under COMESA and EAC could have also impacted national progress in GE regulation. Uganda adopted the COMESA-backed regional policy on biotechnology and biosafety and COMESA Biotechnology and Implementation Plan (COMBIP) to facilitate trade in commodities containing GE ingredients within the common market.

#### 5.1.3 Scientific and technological advances

Uganda has yet to approve the commercial cultivation of GE crops but has made tremendous process in research and development (Table 1). This research was led by local institutions but involved international collaborations (Zawedde et al., 2018). However, human and livestock health products derived from GE technologies, such as vaccines and hormones, have been used in the country for more than four decades. The need to develop national biosafety policy and legislation in Uganda was prompted by an engagement between UNCST and Makerere University in 1992. A team from the Department of Animal Science in the Faculty of Agriculture at Makerere University was proposing to test, in Ugandan cattle, a genetically modified bovine somatotropin (BST) hormone for growth and milk production. The BST hormone was produced through the genetic engineering. In 1993, UNCST received the application for testing the BST, however the review process was interrupted by the controversies over trade in genetically modified organisms between the United States of America and the European Union (EU), which resulted in the EU placing a moratorium on the use of BST in dairy cows in December 1993 (European Council, 1993).

**TABLE 1:** Selected research, development and field-testing activities involving GE products in Uganda.

Sector/Organism	Traits/areas under R&D	Status	Year of approval
Banana	Black sigatoka disease resistance	Confined field trials concluded	2006
	Pro-vitamin A and Iron enhancement	Deregulation trials concluded	2009
	Bacterial wilt resistance	Confined field trials concluded	2010
	Nematode and banana weevil resistance	Confined field trials on-going	2012
Cotton	Bollworm resistance and Roundup herbicide tolerance	Confined field Trials concluded	2008
Maize	Drought tolerance	Confined field trials concluded	2010
	Stem borer pest resistance	Confined field trials concluded	2012
Cassava	Mosaic resistance	Confined field trials concluded	2008
	Brown streak disease resistance	Deregulation trials concluded	2010
Rice	Nitrogen and water use efficiency and salt tolerance	Field trials conducted	2012
Potato	Potato blight resistance	Confined field trials conducted	2015
Sweet potato	Sweet potato virus disease resistance	Confined field trials conducted	2013
Soybean	Glyphosate herbicide tolerance	Confined field trials ongoing	2024
Livestock health	Anti-tick vaccine	De-regulation studies underway	2021
Human health	GM mosquitoes	Contained testing on-going	2022

Source: NBC, official documents.

In 1995, another proposal was submitted to UNCST to conduct Phase 1 clinical trial of a candidate HIV-1 vaccine (ALVAC vCP 205) (Mugerwa et al., 2002). This vaccine was the first preventative HIV-1 vaccine study in Uganda and in Africa and its construct included a live recombinant canarypox vector expressing HIV-1 glycoproteins 120 and 41. These events formed the basis for developing the national biosafety guidelines and the establishment of a National Biosafety Committee (NBC) in 1996 using the Uganda National Council for Science and Technology (UNCST) Act, Cap 211.

In the later half of the 1990s, there was limited research or capacity to test GE products in the country until the turn of the century when the government of Uganda made deliberate investments in, and policy on, building the needed infrastructure and human capacity to use the technology. At the time, when Uganda embarked on development of the National Biosafety Framework for Uganda, with support from United Nation Environment Programme and Global Environment Facility (UNEP and GEF) in the late 90’s key institutions deploying modern biotechnology as a research tool were: Makerere University, National Agricultural Research Organisation (NARO), Uganda Virus Research Institute (UVRI) and Joint Clinical Research Centre (JCRC) from the public sector and Medbiotech Laboratories (MBL) from private sector.

Technology progress within the country’s borders has also shaped the recent development of gene editing guidelines for the country. Research applications for gene-edited rice and cassava prompted the UNCST to develop some guiding principles for review and risk assessment for these newer techniques. Advances in biotechnology, and particularly applications to conduct research and other use activities using new technologies within the country, have been a major trigger for the development and review of safety regulations. This trend is similar in other jurisdictions, including China, European Union, Kenya, United States of America (United States) and Australia, among others (Liang et al., 2022; Turnbull et al., 2021). The array of GE tools available implies that a multitude of different processes and products are possible, and yet each of these techniques may require a different approach to risk assessment. The clearest example is the emergence of the CRISPR/CAS system that may result in genetic changes that cannot be distinguished from their non-GE counterparts.

More recent developments in livestock health, such as NARO’s testing of an anti-tick vaccine (Kabi et al., 2024), have further prompted the review of the biosafety system to improve coordination with other legal entities, such as the National Drug Authority, which is mandated to regulate medical preparations and other pharmaceuticals.

In human health, the opportunity created by CRISPR techniques for gene therapy also prompted further review and integration of human health applications in the biosafety system, despite earlier consensus to exempt pharmaceutical applications such as drugs from direct GE regulation. Owing to the advance in gene therapy research for treatment of sickle cell disease and HIV by the Joint Clinical Research Centre (JCRC) in Uganda, the Office of the Prime Minister is leading other Government Agencies to revise the draft biosafety law to accommodate gene therapy applications for human health (Ityang, 2022; Busiku, 2024). Whereas human and livestock drugs have been under the regulatory purview of the National Drug Authority, scientific breakthroughs such as gene therapy, a form of personalised medicine, required a different approach because it uses genetic engineering of calls and tissues of humans for the treatment of diseases and the long lasting nature of this technology in the body, in many cases life long, impacts on the patient, including changes to the patient’s genome–especially in the organs where genetic changes are made. Gene therapy options may also be tailored to specific patients depending on their unique genetic makeup.

By the end of 2024, biosafety applications received by Uganda National Council for Science and Technology (UNCST) were about 25 including those for research and GM products importation. The NBC has approved over 20 applications for conducting confined field trials and other trials for research and generation of safety-related data (Hafashimana, 2024).

#### 5.1.4 Policy outreach

Biosafety policy reforms in Uganda, particularly related to efforts to develop standalone legislation, have been largely driven by policy outreach, education and public communication on biotechnology and biosafety. Whereas earlier efforts in regulatory discourse focused on key technical stakeholder groups within research and regulatory institutions, there was increased outreach in the past 15 years aimed at obtaining approval (or not by some groups), of a biosafety law. Outreach efforts, which were in many cases divergent, were conducted by government institutions, private sector, individuals, civil society, academic and research institutions, and policy leaders. For example, local academia and researchers contributed to defining the scope of biosafety and biosecurity in Uganda (UNAS, 2010).

**FIGURE 2:**
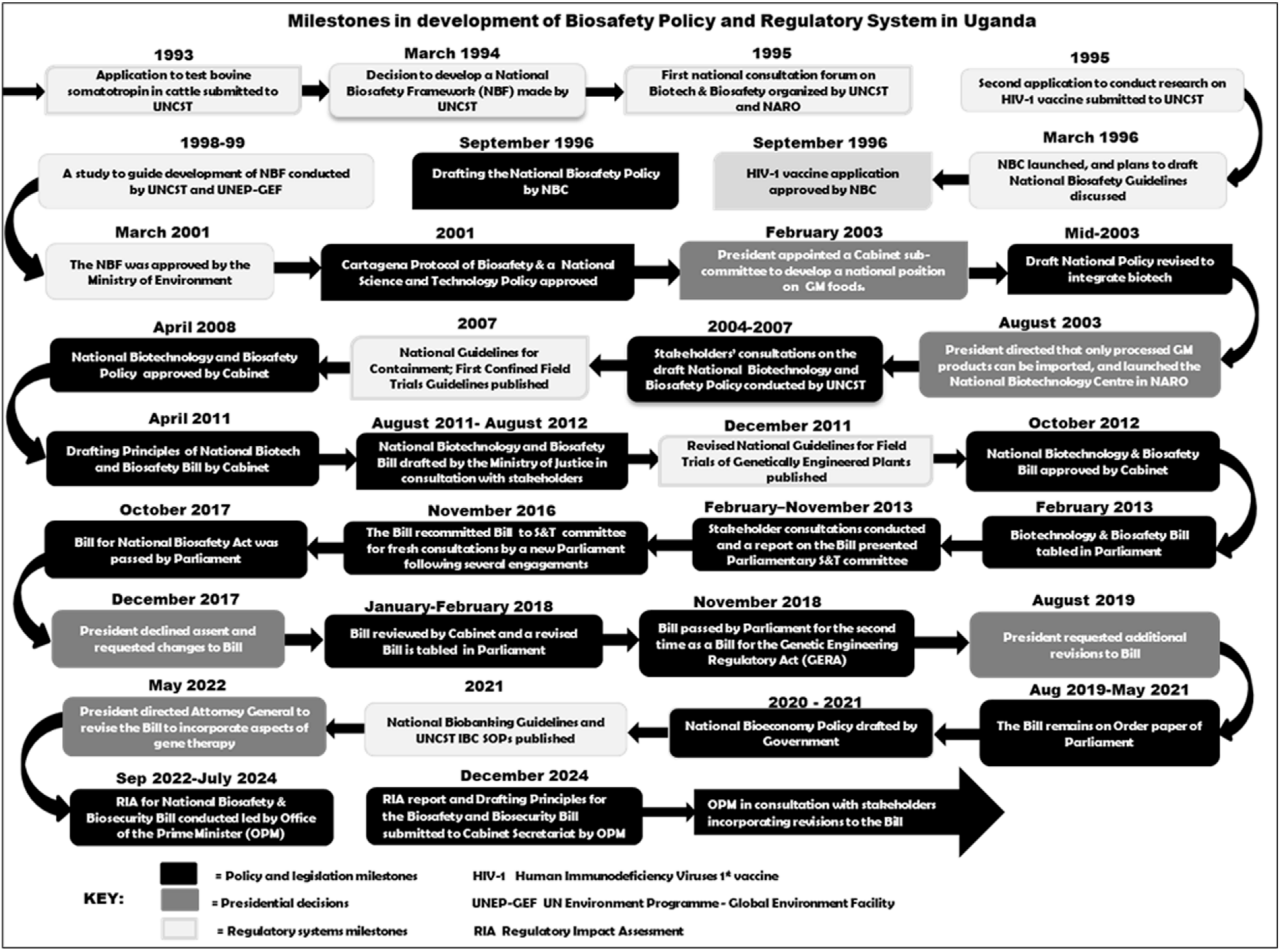
Uganda’s tortuous path in a development of a national policy and law for biotechnology and biosafety.

The National Biotechnology and Biosafety Bill (2012), the first incarnation of a draft law, was drafted largely with technical expertise, based on a process and product risk assessment approach. Scientists and others in academia supported this approach while a section of civil society actors championed more restrictive regulation of the technology (Zawedde et al., 2018; Lukanda, 2020; Busiku, 2024) and demanded amendment to earlier drafts. Outreach against the enactment of the biosafety law impacted the decision by the Executive branch of Government not to approve the bill, after the Parliament passed it twice in 2017 and 2018 (Figure 2). The Executive highlighted many issues commonly raised by selected civil society actors, for example, the concern on potential loss of indigenous biodiversity and the need to label GE products. The current regulatory system is still based on Standard Operating Procedures (SOPs) and guidelines issued by UNCST. These instruments tend to lean towards a more structured risk assessment process for research and development of GE products (UNCST, 2012; 2021).

### 5.2 Key milestones in developing the biosafety system in Uganda

#### 5.2.1 Policy and regulatory milestones

##### 5.2.1.1 Development of a national biosafety framework

Under the guidance of UNCST, in 1994, the country decided to progress by developing a biosafety framework including a policy, regulatory system, public awareness and decision-making process. In 1995, UNCST, in collaboration with NARO organized the first national forum to discuss biotechnology and biosafety and one of the recommendations was to establish a decision-making committee.

During the period 1998–1999, UNCST supported by UNEP-GEF conducted a stakeholders’ consultation study to guide the development of a National Biosafety Framework (NBF) for Uganda. Various stakeholders, including relevant ministries, parliament, the media, public research institutes, academia, and consumer organizations, contributed to conceptualizing the NBF, including the regulatory regime, institutional system, risk assessment and risk management procedures, and an information dissemination system. In March 2001, the Ministry of Environment approved the proposed NBF for Uganda. The approved NBF was a key milestone in biosafety implementation.

##### 5.2.1.2 National biotechnology and biosafety policy

Initially, the country considered developing biosafety regulations under the UNCST Act, however, this option was abandoned when it became clear that this law does not provide adequately for all issues that require regulation, including commercial or environmental release decisions and sanctions for non-compliance. The Government resolved to develop a on a stand-alone policy. The UNCST Statute empowered UNCST to lead the development of the National Biotechnology and Biosafety Policy, which was formally approved by the Uganda Cabinet in 2008 following 6 years of consultations and drafting. The National Biotechnology and Biosafety Policy (2008) underscores the need to harness the benefits of biotechnology while being cognizant of potential risks. The policy also recognizes relevant institutions that would be involved in realizing the objectives of the policy, including UNCST, NEMA, and MAAIF. It further provided terms of reference for NBC and the institutional biosafety committees (IBCs) to ease the work of the NBC, detailed information on risk assessment and management procedures for approval, establishment, and inspection of regulated products.

##### 5.2.1.3 Biosafety regulatory provisions under existing laws

In the past three decades, the government of Uganda has included regulatory provisions for GE technology within sector-specific laws. The Animal Breeding Act of 2001 requires all new animal genetic material to conform to biosafety standards issued by UNCST and the Uganda National Bureau of Standards. The Plant and Seed Act (2006) delegates the responsibility of regulating GE crop seeds to UNCST. The Plant Protection and Health Act, 2016, requires importers of living cultures of genetically modified organisms to obtain an import permit. The National Environment Act 2019 mandates the National Environment Management Authority (NEMA) to develop guidelines and prescribe measures for the management of genetically modified organisms, including risk assessments for general releases and liability and redress mechanisms in the event of damage caused by GE organisms. Whereas the National Drug Policy and Authority Act does not explicitly reference genetic engineering, its broad mandate over all drugs has been used to regulate medical preparations derived from the technology, including hormones such as insulin and vaccines.

##### 5.2.1.4 Biosafety guidelines

Uganda has within the provisions of the UNCST Act, 1990 and related sector laws adopted a series of guidelines to ensure safe and ethical practice in biotechnology research and development. These guidelines play a crucial role in guiding researchers, scientists, and companies involved in biotechnology. Key among these are: Guidelines for Risk Assessment that offer a structured approach to evaluating the potential risks associated with GMOs before their release. This includes assessing the environmental, health, and social impacts of GMOs. The National Guidelines for Containment of GMOs (2007), which set out procedures for safely containing GMOs during research and development, also prevent accidental releases into the environment. National Guidelines for Confined Field Trials (2014) regulate the conduct of field trials for GMOs, ensuring that they are carried out in a controlled manner to prevent unintended environmental consequences. The draft National Guidelines on Genome Editing, although still being finalized, these guidelines aim to regulate the use of genome editing technologies such as CRISPR to ensure they are used responsibly and safely. These guidelines provide essential frameworks for ensuring that research and development in biotechnology are conducted in a way that minimizes risk and maximizes safety.

##### 5.2.1.5 Standard operating procedures (SOPs)

Uganda National Council for Science and Technology has published several SOPs to address processes for receiving and reviewing applications, decision making, inspections, and compliance during laboratory and field experimentation. The SOPs have been revised periodically over the past 20 years, to align with the evolving regulatory landscape for biotechnology. The SOPs currently in use include Guidelines for containment (2006); Field Trial SOPs (2012); NBC SOPs (Revised 2012), Inspection Manual (revised 2021), and SOPs for IBCs (2021). The Guidelines for containment provide for both standard procedures and guidelines. In addition to the SOPs, UNCST further published crop specific SOPs tailored to different GE crop experiments. Specific SOPs were published for GE field research with banana, maize, cassava and cotton.

##### 5.2.1.6 Stand-alone biosafety legislation

The approved policy provided for the development and approval of a standalone law governing biosafety. In April 2011, the Principles for the Biosafety Bill were approved by the cabinet, and the drafting of the Bill started in consultation with various stakeholders. In October 2012, the cabinet approved the National Biotechnology and Biosafety Bill 2012 (Government of Uganda, 2012). In February 2013, the Bill was introduced in Parliament by the Minister of Finance, Planning, and Economic Development. The Bill went through the legislative process, including public consultations by the Parliamentary Standing Committee on Science and Technology, which presented its report in November 2013, recommending adoption of the Bill. The Bill however, lapsed during the term of the 9th Parliament (2011–2016) before being reintroduced in the 11th Parliament (2016–2021). Parliament finally approved the Bill for the National Biosafety Act in October 2017. However, the President of the Republic of Uganda did not assent to the Bill and proposed several changes including changing the name and scope of the proposed law. The key concerns of the President were potential loss of indigenous biodiversity due to adoption of improved varieties/breeds, rights of indigenous owners of genetic resources and liability and redress issues to mention a few.

The Bill for the National Biosafety Act prescribed a unified approach to regulating biotechnology across various sectors, including agriculture, industrial applications, and human health. The Bill addressed key areas such as: risk assessments - ensuring that any GMO introduced into the environment or food system is thoroughly evaluated for potential risks to human health, biodiversity, and ecosystems; -public participation -mandating that public consultations and hearings be conducted before general release approvals, allowing stakeholders, including farmers, consumers, and environmental groups, to provide feedback; monitoring and compliance -establishing mechanisms for ongoing monitoring of GMOs in the field to ensure they do not cause unintended harm; appeal processes; and liability and redress mechanisms. Consistent with the Cartagena Protocol on Biosafety, the Bill had designated a Directorate responsible for biosafety within the then Ministry of Science, Technology and Innovation as the Competent National Authority (Komen, 2000; Masiga, 2015).

Following the President’s decision, the Government and Parliament revised the Bill for National Biosafety Act to the Bill for the Genetic Engineering Regulatory Act (GERA) in early 2018. The new bill was subsequently passed by Parliament in November 2018. The President again declined assent to this version of the bill through a letter to Parliament in August 2019, requesting some further changes including clauses to address separation of GE and Non-GE materials and labelling for identification of GE products. The 2016–2021 Parliamentary term lapsed without further debate on the bill despite being included on the order paper consistently. The Uganda Constitution requires a two-thirds majority vote of all members of Parliament to approve a bill returned twice by the President. It was unlikely this would be achieved.

Following a directive of the President issued in 2022, the Government of Uganda, through the Office of the Prime Minister and office of the President restarted the drafting process for a new bill, to also take on board GM applications to Human health, which had not been addressed in the previous bill since they had not yet gained prominence at the time of drafting (Ityang, 2022). A regulatory impact assessment was conducted in 2023–2024 with leadership of the Prime Minister and principles of the bill prepared. A formal bill is expected to be drafted and approved by the Cabinet once the principles and RIA report are adopted. Numerous milestones have contributed to the development of biosafety policy and regulatory systems in Uganda (Figure 2).

#### 5.2.2 Institutional development milestones

##### 5.2.2.1 Institutional mechanisms and regulatory bodies

The effective implementation of Uganda’s biosafety system relies on the coordination and collaboration of several institutions. Uganda’s Biosafety institutional development mirrors the Cartagena’s framework of having: Biosafety Clearing House (BCH), Advance Informed Agreement (AIA), Decision-making process, Meeting of the Parties, Technical Advisory Bodies. The institutional milestones focus on new institutions or realignment to existing institutions in response to the CBD and attendant protocols. The institutions include both policy and technology development categorized as Ministries, Departments/Directorates and Agencies (MDA’s) such as: the Ministry responsible for Science Technology and Innovation, Ministry of Water and Environment (MWE), Ministry of Finance, Planning and Economic Development (MoFPED), Ministry of Agriculture, Animal Industry and Fisheries (MAAIF), Ministry of Trade, Ministry of Health, Ministry of Education, Uganda National Council for Science and Technology, Science Technology and Innovation Secretariat - Office of the President, National Environment Management Authority, Uganda National Drug Authority, Uganda National Bureau of Standards, and various sectoral National Research organisations (in Agriculture, human health, animal health, wild life) (Table 2). The institutional development milestones highlight human and infrastructure capacity development related to ensuring Biosafety of gene technology in Uganda.

**TABLE 2:** Institutional roles and milestones.

Institution	Biosafety role	Legal mechanisms	Date biosafety functions commenced
Policy and regulatory			
UNCST	Competent National Authority	Law; Policy	1992
NBC	Technical Advisor to the competent authority	SOP, guidelines	1996
MWE	National Focal Point; Biosafety Clearing House	Policy	2003
MAAIF	Import approval for living cultures of plant, microbial and animal products; biosafety inspections	Law; Policy	2006
NEMA	Environmental Risk Assessments; Liability and redress	Law	2019
Ministry of Trade, Industry and Cooperatives (MTIC)	Standards enforcements (UNBS)	Regulations	2022
Ministry of Science, Technology and Innovation (MoSTI) (2016–2021)	Biosafety policy oversight	Policy	2016
STI Secretariat in Office of the President	Science policy and R&D financing	Policy	2021
Institutional Biosafety Committee (IBCs)	Internal compliance enforcement	SOPs; Guidelines; Policy	2007
Technology development			
NARO	Agricultural regulatory compliance during R&D	Act	2005
Health research agencies: Uganda Virus Research Institute (UVRI), Uganda Joint Clinical Research Center (JCRC) and Uganda National Health Research Organisation (UNHRO)	Health regulatory compliance during R&D	Policy	2011
Universities	Regulatory compliance during R&D; human capacity development in biosafety	Policy	1993

#### 5.2.3 Developments in public engagements

##### 5.2.3.1 Outreach efforts

A key strategic focus of the NBC and UNCST has been the promotion of public engagement and awareness regarding the benefits and risks of modern biotechnology. Recognizing that successful biotechnology governance depends on informed decision-making, the NBC has adopted a comprehensive approach to engaging various stakeholders, including policymakers, scientists, farmers, civil society organizations, and the public. The NBC has spearheaded initiatives aimed at institutionalisation of Biosafety engagement among stakeholder institutions through such mechanisms as: the National Biotechnology and Biosafety Communication Strategy and the Annual National Biosafety Forums.

The National Biosafety Forums, provide a platform for open discussions on critical policy issues related to biotechnology since 2016. These forums, which have attracted over 1,200 participants from various sectors, have provided a platform for key stakeholders to engage in dialogue, share perspectives, and collaboratively address concerns surrounding the use of GMOs. The forums have become an essential tool in fostering a shared understanding of the potential benefits and challenges posed by modern biotechnology, thereby enhancing transparency in policymaking. Additionally, the NBC has implemented public education campaigns aimed at dispelling myths and misconceptions about GMOs. By leveraging media outlets, public outreach programs, and community engagement initiatives, the NBC has actively worked to build trust in biotechnology regulation, ensuring that the public remains well-informed and empowered to participate in the ongoing national discourse on biotechnology and biosafety.

The NBB Policy (2008) also emphasizes public awareness, with the aim of dispelling myths, fostering an informed discourse, and addressing societal concerns. Sensitization of the Ugandan public, policymakers, and stakeholders are well-informed about the benefits and risks of biotechnology is crucial to gaining broad public trust in the regulatory system. Public education campaigns have been carried out using radio and television talk shows, print media articles and social media messages. Communication and sensitisation efforts were conducted by different institutions in the public, civil society and academia.

##### 5.2.3.2 Building relationships among regulators, scientists, media and the public

Ugandan scientists have been working closely with UNCST and NBC to ensure that the research in modern biotechnology aligns with the national regulatory requirements that provides for conducting biotechnology research while ensuring safety for humans, animals, and the environment. Scientists in Uganda have been and continue to collaborate with regulators to build capacity for biosafety assessment through training workshops, seminars, and educational programs on biotechnology and biosafety even at institutions of higher learning to enhance understanding of biosafety protocols on good scientific practices when developing or manipulating biotechnologies, in partnerships with international organizations and universities. UNCST was instrumental in establishing panels of experts and professionals such as the Uganda Biotechnology and Biosafety Consortium (UBBC) and the Biosafety and Biosecurity Association of Uganda (BBA-U), coalitions of policymakers, scientists, private sector actors and the media established in the 2000’s to advance the role of biotechnology in improving livelihoods and ensure the safety its application.

Scientists with an aim of building trust and transparency in the regulatory process have through available communication systems built capacity in the communication of biotechnology and biosafety through engaging the public and key stakeholders in public consultations, media campaigns, and stakeholder workshops to demystify biotechnology and address biosafety concerns, which validates the need for even better communication systems that will ensure compliance with the biosafety requirements by scientists at local and international markets.

Scientists have continued to cooperate with regulators in monitoring and evaluation by providing detailed records, complying with biosafety conditions, and allowing access for evaluation during rigorous inspections and audits of research facilities and field trials, supporting responsible research and development while ensuring compliance with international biosafety standards, such as the Cartagena Protocol on Biosafety and its supplementary Protocol on Liability and Redress, by working with global regulatory bodies facilitating international partnerships.

Scientists in Uganda undertake research on risk assessment to assess and mitigate potential risks associated with biotechnology products, share their data with regulatory authorities to inform decision-making and enhance biosafety measures but also provide support for policy development through contributing to policy discussions by providing evidence-based insights to regulators to ensure that biosafety policies are grounded in scientific understanding and practical realities.

#### 5.2.4 Milestones in international and regional partnerships

Within its mandate as the Competent Authority, the UNCST has participated in regional and international biosafety collaborations and partnerships. The main focus of the partnerships included: a) national capacity building for risk assessment and decision making; b) infrastructure development for biotechnology and biosafety; and c) regional harmonisation of policies (Komen, 2000).

Regionally, Uganda took an active role in promoting biosafety engagement with other countries and communicating biosafety. The regional partnerships, supported under several different initiatives, were instrumental in developing harmonised policies within the East African Community and the Common Market for Eastern and Southern Africa. These partnerships aimed to facilitate trade in products and exchange of genetic resources within these common markets. They further supported the early development of national policies and ensuring the needed alignment with international biosafety frameworks. Capacity development milestones achieved through regional and international partnerships included the training of more than 1,000 Ugandan scientists, regulators, policymakers in biosafety risk assessment and management and in biosafety communication.

## 6 Lessons learnt and their implications for national biosafety systems discourses

The journey of establishing and strengthening national biosafety systems in Uganda has provided valuable insights that can guide future policy development and strategic implementation in biosafety regulation. Several critical lessons have emerged, which if addressed proactively can help shape more effective, adaptive, and resilient biosafety systems globally. These lessons not only inform national approaches but also contribute to regional and international efforts.

### 6.1 Lessons learnt

#### 6.1.1 Building and sustaining capacity for all relevant regulatory agencies is key for effective implementation

Having relevant laws and policies is not enough, similarly focusing capacity building efforts to a few key regulatory agencies is inadequate. We must consistently strengthen capacity in all relevant regulatory agencies like UNCST, STI-OP, Ministry of Health and its agencies, Ministry of Trade and Industry, UNBS, Ministry of Justice, MWE, MAAIF and NEMA to enforce regulations, conduct risk assessments, and monitor GMOs use. This can be achieved by ensuring that there is adequate funding, training personnel, infrastructure and increasing coordination among agencies that are tasked with effective biosafety governance. The rapidly evolving nature of biotechnology necessitates that regulatory bodies/agencies continuously update their capacities according to scientific developments and best practices in biosafety. National investment in training and the development of specialized expertise within these institutions and supporting relevant infrastructural capacity is critical for effective biosafety regulation.

#### 6.1.2 There is need for flexibility and adaptability in biosafety regulation

In absence of an overarching law for biosafety, researchers, investors and policymakers need to adapt to using available existing laws and regulations to support development and application of biotechnology that is rapidly advancing. For example, new scientific advances like gene editing techniques require the adoption of progressive regulatory approaches to keep up with these developments. Biotechnology is a global enterprise, and the risks and benefits associated with its use transcend national borders. In addition, international collaboration and alignment with advances in global biosafety standards also influence biosafety systems. Countries should actively participate in international biosafety initiatives, share experiences, and adopt best practices to promote responsible and safe biotechnology applications. All the above, require that biosafety regulatory systems should be flexible and adaptable, while at the same time protecting national interests.

#### 6.1.3 There is need to balance innovation with risk management

Uganda’s experience highlights the need to balance potential risks and benefits of biotechnology as demonstrated by the effective systems in African countries such as South Africa and Kenya that support agricultural innovation while addressing biosafety concerns. Rigorous and transparent risk assessment remains a cornerstone for regulation of modern biotechnology innovations. Development of appropriate risk management plans to ensure that approved products or technologies for development, release into the environment and onto the market will be effectively regulated.

Risk management plans should be data-driven (with provisions for review of decisions, in case new data become available), transparent, and inclusive of all potential acceptable options and their implications. International cooperation in sharing risk assessment and risk management data and methodologies can also improve the consistency and quality of evaluations and management plans.

#### 6.1.4 Consistent public engagement is critical for building trust

The GMO debate is highly polarized, despite continuous capacity-building that was done by different organizations and the media. Concerns focus on food safety, farmer dependence on biotechnology companies, and the loss of indigenous seed varieties continue to come up. Public perception of modern biotechnology plays a central role in the success of biosafety governance. Effective public engagement and transparency are therefore essential to ensure that communities are informed about the benefits, regulatory processes and risk management plans, where necessary, related to modern biotechnologies. Public opposition to GMOs in Uganda is driven by misinformation, fear of corporate control over seed and ethical concerns.

We have learnt that biosafety discussions are successful when farmers, consumers, scientists, civil society and the media are engaged to show that every decision made is informed by scientific evidence and incorporates socio-economic considerations of the receiving community. This has prompted the proposed Uganda’s biosafety decision making process to be inclusive in nature (Zawedde et al., 2018). In the era of social media and AI, the public and policymakers are understandably lost in the frenzy of sensational and animated misinformation therefore regulators should also tap into this era to demonstrate transparency of the biosafety system.

Future national biosafety systems discourses must incorporate consistent public engagement (in line with the CPB Article 23) and transparent risk communication to build trust in the biosafety regulations. This requires tapping into various effective communication strategies depending on the target audience. Therefore, a substantial budget must be allocated for consistent engagement of diverse stakeholders. In addition, regulatory bodies must commit to implement transparent decision-making processes.

#### 6.1.5 Ensuring accountability and robust monitoring and enforcement mechanisms

Establishing biosafety regulatory system is not sufficient on its own, accountability and enforcement are vital to ensure compliance with biosafety standards and the protection of human health and the environment. Rigorous monitoring systems that track modern biotechnology applications, from research to commercialization and post-release monitoring, should be integrated into regulatory systems. Regular inspections, audits, and follow-up assessments will ensure that biosafety standards are maintained throughout the lifecycle of modern biotechnology projects, as well as build public and stakeholders’ trust in the regulatory system.

Government investment in comprehensive monitoring and enforcement mechanisms to hold organizations and researchers accountable for adhering to biosafety protocols is pertinent. This includes building capacity for monitoring and improving traceability systems in relevant regulatory bodies and introducing corrective actions and penalties for non-compliance in regulations.

### 6.2 Strategic recommendations

As Uganda continues to build on its biosafety regulatory system, several future-oriented actions are necessary to ensure that the country remains a competitive and responsible player in modern biotechnology application.

#### 6.2.1 Further investment in cutting-edge technologies

Uganda must invest adequate resources in advanced biosafety and biotechnology research tools and bioinformatics platforms to stay at the forefront of global biotechnology trends such as synthetic biology, gene editing, gene drives and artificial intelligence in research. This investment will ensure that Uganda can continue to safely innovate in these emerging fields while maintaining rigorous safety standards especially for products that are potentially entering the country.

#### 6.2.2 Regional harmonization in biosafety regulation

Uganda should continue to strengthen its role in regional collaboration in biotechnology governance, particularly by advocating for and supporting the development of a harmonized East African biosafety regulatory framework. This will enhance the free movement of biotechnology innovations across the region, creating opportunities for joint research initiatives, biotechnology trade, and mutual recognition and enforcement of biosafety standards.

#### 6.2.3 Strengthening public-private partnerships (PPPs)

To accelerate the commercialization of biotechnology innovations in Uganda, public-private partnerships (PPPs) should be fostered to build collaboration between the government, research institutions (supported by a strong legal well-informed team of lawyers, to ensure fair and mutually beneficial terms of engagement), and the private sector. This would ensure the safe and efficient transition of innovations from laboratories to market-ready products, with well protected and mutually beneficial IPR systems.

#### 6.2.4 Sustainable infrastructure development

Investment in biosafety laboratory infrastructure, especially at border entry points is essential to expand Uganda’s capacity for biosafety testing and food safety assessments. Additionally, partnerships with international laboratories can help accelerate the development of local human capacity and tapping in existing infrastructure elsewhere to support large-scale biotechnology commercialization.

## 7 Conclusion

Modern biotechnology is evolving rapidly, and its application is increasingly used in various sectors either through local research and development or importations. Uganda’s experience in biosafety system development underscores the need for flexible, inclusive, and scientific evidence-driven regulations, which will be achieved by addressing public concerns, strengthening institutions capacity, and aligning policies with regional and global standards. Uganda can create a national biosafety system that supports innovation while ensuring safety to the environmental and human/animal health. This will shape scientific innovation, promote climate resilience and environmental sustainability, grow trade opportunities while uploading public trust and protecting national interests. A coordinated, flexible and evidence-based regulatory system is essential for effective application and adoption of the current and emerging modern biotechnologies to support the country’s economic, food and nutrition security.
